# Some Rarer Forms of Nephritis

**Published:** 1904-10-01

**Authors:** 


### SOME RARER FORMS OF NEPHRITIS.

In his Croonian lectures Dr. J. Rose Bradford[1] alludes to some of the less common varieties of nephritis. Apart from gummata and amyloid degeneration of the kidney which may occur in the late stages of syphilis, a nephritis is not very uncommon within two years or so of the date of specific infection. This is difficult to distinguish from other varieties of nephritis and unless the possibility of syphilis is borne in mind it may, when it occurs, be regarded as the result of exposure to cold or wet. But there are one or two clinical features which may help towards a correct diagnosis. Thus the amount of albumen is very large, the urine even becoming practically solid on boiling. Yet with this there is not, as in so many other cases of nephritis with marked albuminuria, anything like extreme diminution of the quantity of the urine ; nor are casts very abundant, and casts with definite renal elements are extremely scanty. Apart from the albuminuria there is little in the patient's general condition to suggest that he is the subject of kidney disease, and in particular dropsy may be completely absent. Milk diet, it has been noted in these cases, produces but little improvement, and has no sensible effect on the amount of albumen. The albuminuria may persist for many months, and yet in the end completely disappear. On the other hand, the development of uræmia is possible, and this may prove fatal. Though renal disease is often held to be a contra-indication to the use of mercury, antisyphilitic remedies in the cases here referred to prove very successful.

Another form of renal disease may be named from its pathological anatomy "small white kidney." This is to be distinguished from the contracted or small red kidney which is found in the form of chronic Bright's disease most frequently seen in male adults who have reached middle life. And the grounds of distinction are clinical as well as pathological. The patients are generally about 25 to 30 years of age, and the disease is apt to run a latent course, so that no symptom of it may be observed until a few weeks before death, which may be preceded by acute uræmic manifestations. General loss of strength, wasting, pigmentation of the skin, headache, failure of sight from retinal changes, are symptoms that may first attract the patient's attention. In other instances nothing is noted until definitely uræmic phenomena appear. An attack of acute mania has been known as the first evidence of the disease, and cataleptic phenomena and dyspnœa (regarded as asthma) have been found to have a similar explanation. Further, in this form of renal disease, whilst (as in the true granular kidney) the quantity of urine is increased rather than diminished, and the specific gravity is low, the amount of albumen is not slight or scanty, but is indeed considerable, amounting to a third, or even a half. Casts of the hyaline and granular variety are found, but they are not always numerous. The danger of the condition is the onset of uræmia, which often develops rapidly, and has an extremely serious prognosis; it is much more fatal than in the acute forms of kidney disease. According to Dr. Bradford, the contracted white kidney is a distinct pathological and clinical entity, and is not a late result of the large white kidney (chronic parenchymatous nephritis).

[1] Lancet, July 16th, 1904, *et seq.*

